# Neighborhood economic and demographic landscape as predictors of 90-day outcomes post-stroke hospitalization

**DOI:** 10.3389/fstro.2026.1738822

**Published:** 2026-03-12

**Authors:** Farya Fakoori, Karlon H. Johnson, Hannah Gardener, Carolina M. Gutierrez, Negar Asdaghi, Lauri Bishop, Scott C. Brown, Iszet Campo-Bustillo, Gillian Gordon Perue, Emir Veledar, Hao Ying, Lili Zhou, Jose G. Romano, Tatjana Rundek, Erika Marulanda

**Affiliations:** 1 University of Miami Miller School of Medicine, Miami, FL, United States; 2 Department of Neurology, Evelyn F. McKnight Brain Institute, University of Miami Miller School of Medicine, Miami, FL, United States

**Keywords:** community health, neighborhood SES, stroke outcomes, stroke readmission, transitions of care

## Abstract

**Objective:**

An in-depth exploration of neighborhood environmental impact on post-discharge stroke outcomes is lacking yet essential for identifying populations at high risk. We assess neighborhood economic and demographic characteristics associated with 90-day death or readmission post-stroke hospitalization.

**Methods:**

We prospectively analyzed 1,329 acute stroke survivors in the Florida Stroke Registry's Transition of Care Stroke Disparities Study (91% ischemic, 56% male, 52% non-Hispanic White, 23% non-Hispanic Black, 22% Hispanic, median age 64). Neighborhood characteristics at the ZIP+4 level, including socioeconomic status (NSES), racial/ethnic composition, and business densities (food, tobacco/alcohol, gyms, medical services), were analyzed using factor analysis to generate four factors with eigenvalues greater than 1. Outcomes (death or readmission) were assessed through structured telephone interviews 90 days post-discharge. Logistic regression evaluated associations between neighborhood characteristics and outcomes, adjusting for demographics (race/ethnicity, sex, age), vascular risk factors, stroke severity from Get With The Guidelines-Stroke^®^, and social or economic conditions such as insurance, support, and living arrangements.

**Results:**

Within 90 days, 208 patients experienced death or readmission. Four factors explained 59% of the variance in 24 neighborhood characteristics. Factor 1, defined by lower NSES, higher population density, and urbanization (RUCA code 1, greater densities of tobacco/alcohol outlets, restaurants, grocery stores, gyms, and pharmacies), was associated with a 20% increased risk.

**Conclusions:**

Living in densely populated, highly urbanized neighborhoods with lower SES and greater commercial density predicted poor stroke outcomes independent of individual health or SES. These findings can guide community interventions to reduce stroke mortality and readmission.

## Introduction

Stroke remains a leading cause of disability and death, particularly among older adults (Tsao et al., 2022), and significantly contributes to years of healthy life lost due to disability and premature mortality in middle-aged and older Americans (McGrath et al., 2019). While individual characteristics and acute care have been extensively studied as predictors of stroke mortality and readmission (Liu et al., 2024; Morton et al., 2022; Kumar et al., 2022; Pallisgaard et al., 2020; Nkemdirim Okere et al., 2020; Man et al., 2020; Bjerkreim et al., 2019; Nouh et al., 2017), the influence of neighborhood-level factors on these outcomes remains less understood. This knowledge gap persists due to the variability and interrelated nature of neighborhood characteristics and their interactions with individual patient factors.

Current research suggests that higher neighborhood socioeconomic status (SES) is associated with better stroke outcomes. Stroke patients from higher SES neighborhoods are more likely to receive superior in-hospital care (Kim et al., 2023; Forman et al., 2024), such as faster hospital arrival and timely mechanical thrombectomy for ischemic stroke, and experience improved post-discharge outcomes, including lower mortality rates (Yu et al., 2021), better functional recovery (Delhey et al., 2024; Cote et al., 2024; Stulberg et al., 2021; Twardzik et al., 2019), and lower healthcare costs post- discharge (Yu et al., 2021). In addition to SES, environmental features such as walkability, green spaces, and lower air pollution (Liao et al., 2022; Lang et al., 2022; Yitshak-Sade et al., 2019) are linked to improved cardiovascular health and survival. Demographic factors, including a lower proportion of older adults and non-Hispanic Black residents in a neighborhood, have also been associated with better health outcomes (Hu et al., 2021; Ji et al., 2020; Hu et al., 2020). However, most studies have explored these factors in isolation, lacking a comprehensive analysis of how neighborhood characteristics collectively influence stroke outcomes.

This study addresses the need for an integrated evaluation of neighborhood influences by simultaneously examining the effects of multiple neighborhood social and built environmental characteristics such as urbanization, socioeconomic status, and racial composition on 90-day mortality and readmission rates following stroke hospitalization. By considering the combined impact of these factors, we aim to provide a nuanced understanding of how neighborhood environments shape stroke recovery and inform strategies for targeted community interventions.

## Methods

Funded by the National Institutes of Health (NCT03452813), the Transitions of Care Stroke Disparities Study (TCSD-S) is a prospective study designed to identify disparities in stroke care transitions following hospitalization, focusing on gender, racial/ethnic, and regional differences (Johnson et al., 2023). TCSD-S is a subset of the Florida Stroke Registry (FSR), which includes 180 hospitals across Florida (Marulanda et al., 2023; Asdaghi et al., 2023). All FSR hospitals utilize the American Heart Association's Get With The Guidelines-Stroke (GWTG-S) tool to collect standardized data on acute stroke care. The TCSD-S participating hospitals were intentionally selected *a priori* to capture geographic, demographic, and healthcare delivery diversity across Florida and included 10 Comprehensive Stroke Centers distributed across North, Central, and South Florida. These sites represent a mix of university-affiliated and large community-based hospitals and serve broad catchment areas encompassing urban, suburban, and rural populations with substantial socioeconomic heterogeneity. The 10 TCSD-S hospitals further extend this by conducting structured telephone interviews at 30 and 90 days after discharge to collect information on post-discharge behaviors and outcomes. Assessments focus on behavioral modifications, including medication adherence, dietary changes, exercise adherence, medical and rehabilitation follow-ups, and cessation of harmful substances (e.g., tobacco, alcohol, and marijuana) as well as stroke outcomes, including readmission, emergency room visits, stroke recurrence, cardiovascular events, stroke-related disability, and death.

The study protocol was approved by the University of Miami Institutional Review Board (IRB). Written informed consent was obtained from all participants prior to hospital discharge.

### Data availability statement

The Florida Stroke Registry (FSR) utilizes data from Get With The Guidelines-Stroke^®^ (GWTG-S), collected primarily for quality improvement. Researchers seeking access to this data must submit a research proposal through http://www.heart.org/qualityresearch. Proposals are reviewed by the GWTG-S and FSR advisory and publication committees upon reasonable request.

### Study sample

From the original 1,416 TCSD-S enrollees (2018–2023), 1,330 patients with complete 30- and 90-day follow-up records were identified. After excluding one patient of unknown sex, the final analysis included 1,329 patients with a confirmed diagnosis of ischemic or hemorrhagic stroke, discharged to home or inpatient rehabilitation, and complete data on 30-day behavioral modifications.

### Data collection

#### Independent variables

Neighborhood characteristics consisting of 24 variables (Table 1) were obtained by Sciera™, a third-party company, using publicly available datasets linked to patients' ZIP+4 codes. These data included neighborhood race/ethnic composition (% Black, % Hispanic, % White), socioeconomic indicators (% below poverty, employment, education, and income levels), Rural-Urban Commuting Area (RUCA) codes (USDA ERS, 2024), population and business densities, and healthcare provider densities (e.g., hospitals, clinics, pharmacies).

**Table 1:** Principal component analysis of the 24 neighborhood characteristics^a^.

**Neighborhood Characteristics**	**Factor 1**	**Factor 2**	**Factor 3**	**Factor 4**
Median % Black	16	−59^*^	−1	4
Median % Hispanic	66^*^	−23	44^*^	−19
Median % below poverty	32^*^	−72^*^	−34^*^	0
Median household income	−28	67^*^	50^*^	−7
Unemployment	19	−71^*^	−32^*^	−9
Median % with bachelor's degree	−7	76^*^	22	11
Total population	51^*^	−11	49^*^	9
Median tobacco density	80^*^	26	−18	3
Median alcohol density	63^*^	33^*^	−22	0
Median restaurant density	82^*^	23	−20	8
Median fast-food density	48^*^	41^*^	−14	24
Median grocery density	74^*^	11	−25	−10
Median pharmacy density	78^*^	21	−7	6
Median gym density	62^*^	45^*^	−15	3
Rehabilitation center (count)	13	18	−10	44^*^
Hospital (count)	26	5	0	55^*^
Median clinic density	58^*^	10	−6	35^*^
RUCA 1	54^*^	23	5	−38^*^
North	−53^*^	−32^*^	−7	56^*^
Panhandle	−15	3	7	6
South	64^*^	−21	61^*^	−2
West Central	−17	44^*^	−56^*^	−43^*^
Crowding^b^	64^*^	−49^*^	42^*^	−12
Housing^c^	−69^*^	41^*^	21	−1

^a^Printed loading values are multiplied by 100 and rounded to the nearest integer. Absolute values greater than 0.3 are flagged by an “^*^”.

^b^Crowding (zip code density measure) = (total housing population)/(count of housing units/median rooms) where Total Housing Population is the total number of people living in owner or renter occupied housing in a zip code.

^c^Housing = (total housing population owner occupied)/(total housing population).

#### Dependent variables

Ninety-day outcomes were collected by the 10 TCSD-S participating hospitals in Florida through structured telephone interviews with patients or caregivers. These interviews captured data on adherence to behavioral modifications related to transitions of stroke care within 30 days post-discharge, as well as 90-day outcomes including re-admissions and mortality.

#### Covariates

Individual patient data were sourced from the Florida Stroke Registry (FSR) using the GWTG-S^®^ tool. These included demographics (age, gender, race/ethnicity), insurance coverage (private, Medicare/Medicaid, or uninsured), and clinical characteristics of the index stroke, such as stroke type, modified Rankin Scale (mRS) score, discharge location (rehabilitation facility vs. home), and stroke severity (National Institutes of Health Stroke Scale, NIHSS).

### Statistical modeling

To simplify the data into fewer dimensions, we conducted an exploratory factor analysis (EFA) to identify the underlying structure of our set of neighborhood variables (Table 1). After examining the distribution patterns of the 24 neighborhood variables, we observed that most were skewed across ZIP+4 codes. Consequently, most of the variables were dichotomized for the EFA as follows:

Race/ethnic composition: neighborhood percentages of Black and Hispanic residents were dichotomized at the median. Neighborhood socioeconomic status (NSES): most variables, including the percentage of residents below poverty, percentage with at least a bachelor's degree, and median household income, were dichotomized at the median. However, total population and unemployment percentage were treated as continuous variables due to their normal distributions. Business and healthcare facility densities: these variables were dichotomized at the median, except for the number of hospitals and rehabilitation centers, which were dichotomized into neighborhoods with no facilities vs. those with at least one (0 vs. ≥1). RUCA score: an indicator of neighborhood urbanization, dichotomized as RUCA score 1 (most urbanized) vs. all others. Florida regions: dichotomized by location (e.g., ZIP code in the south vs. not). Crowding and housing variables: crowding was calculated as the ZIP code density unit (see Table 1), while the housing variable indicated the ratio of homeowners to the population in each ZIP code.

The EFA procedure assigns an eigenvalue to each factor that corresponds to the total variance in item responses explained by each factor. Factors with eigenvalues greater than 1 are usually retained. In this EFA, although six factors had eigenvalues greater than 1, we retained only four factors in our final model. This decision was made iteratively based on several considerations including Scree plot inspection (Supplementary Figure 1): the eigenvalues plateau after the fourth factor, suggesting that subsequent factors capture less distinct information, and most of the variance (59%) in the 24 neighborhood characteristics was explained by the first four factors. “Loadings” (coefficients indicating the strength and direction of the relationship between variables and factors) with values > |0.3| were flagged, indicating significant contributions to the respective factor.

The four main factors identified were used as independent variables in sequential logistic regression models with two levels of adjustment, to assess their associations with a composite outcome of either mortality or readmission within 90-day post-stroke hospitalization. Odds ratios and confidence intervals were calculated, adjusting for individual characteristics in the following steps: Model 1: adjusted for individual demographics (age, sex, race/ethnicity) and socioeconomic factors (living arrangements, social support size, health insurance type, and education). Model 2: included all variables from Model 1 and additionally adjusted for comorbidities, including heart failure, chronic renal insufficiency, prior stroke, atrial fibrillation, hypertension, diabetes, dyslipidemia, smoking history, discharge destination (home vs. rehab), and stroke severity, measured by the National Institutes of Health Stroke Scale (NIHSS), which was modeled using four categories reflecting clinically meaningful strata: minor (0–4), moderate (5–15), moderate-to-severe (16–20), and severe (≥21).

## Results

Out of six factors with eigenvalues greater than 1.0 (Supplementary Figure 1), the first four were retained, explaining approximately 59% of the variance in the 24 neighborhood characteristics (Factor 1: 28%, Factor 2: 17%, Factor 3: 9%, Factor 4: 6%). As shown in Table 1, Factor 1 represents neighborhoods with lower NSES (higher % below poverty, lower home ownership), higher population density, and greater urbanization (business density) with a higher proportion of Hispanic residents reflecting Florida's demographic context; Factor 2 indicates neighborhoods with lower percentages of Black and Hispanic residents, higher NSES (lower % below poverty, higher % educated residents, higher income, less population densities, and more house ownership), and lower business densities; Factor 3 represents Hispanic-dominant, population-dense neighborhoods with high socioeconomic status and low urbanization, and finally, Factor 4 is an indicator of neighborhoods with better access to healthcare facilities.

Table 2 provides a detailed description of individual characteristics and their associations with 90-day mortality and readmission, offering a comprehensive view of the cohort.

**Table 2:** Death or readmission within 90-days post-stroke hospitalization.

**Variable**	**Total (*N* = 1,329)**	**No (*N* = 1,121)**	**Yes (*N* = 208)**	***p*-value**
**Individual characteristics**				
**Sex**				
Male	747 (56%)	635 (57%)	112 (54%)	0.45
Female	582 (44%)	486 (43%)	96 (46%)	
**Age**				
Mean (SD)	64 (13.9)	64 (13.8)	63 (14.3)	0.22
**Race**				
White	680 (51%)	578 (52%)	102 (49%)	0.16
Black	301 (23%)	248 (22%)	53 (25%)	
Hispanic	295 (22%)	255 (23%)	40 (19%)	
Other	53 (4%)	40 (6%)	13 (6%)	
**Lives with**				
Alone	300 (23%)	253 (23%)	47 (23%)	0.86
Spouse/partner	712 (54%)	604 (54%)	108 (54%)	
Children	167 (13%)	141 (13%)	26 (12.5%)	
Other	150 (11%)	123 (11%)	27 (13%)	
**Feels close to**				
Less than 3 people	275 (21%)	223 (20%)	52 (25%)	0.09
3 or more people	1,054 (79%)	898 (80%)	156 (75%)	
**Foreign born**				
Yes	404 (30%)	337 (30%)	67 (32%)	0.54
No	925 (70%)	784 (70%)	141 (68%)	
**Speaks English**				
Yes	1,055 (79%)	884 (79%)	171 (82%)	0.27
No	274 (21%)	237 (21%)	37 (18%)	
**Education**				
Less than high school	170 (13%)	142 (13%)	28 (13%)	0.89
Completed high school	452 (34%)	384 (34%)	68 (33%)	
Some college or more	707 (53%)	595 (53%)	112 (54%)	
**Discharge status**				
Home	1,000 (75%)	858 (76.5%)	0142 (68%)	0.019
Hospice – healthcare facility	1 (00.08%)	1 (00.09%)	0 (00.00%)	
Acute care facility	10 (1%)	8 (1%)	2 (1%)	
Other healthcare facility	298 (22%)	241 (21.5%)	57 (27%)	
Expired	3 (00.23%)	1 (00.09%)	2 (1%)	
Left against medical advice	17 (1%)	12 (1%)	5 (2%)	
**Insurance**				
Private	296 (22%)	259 (23%)	37 (18%)	0.12
Medicare	593 (45%)	506 (45%)	87 (42%)	
Medicaid	66 (5%)	54 (5%)	12 (6%)	
Self/un-insured	371 (28%)	299 (27%)	72 (35%)	
Missing	3 (00.23%)	3 (00.27%)	0 (00.00%)	
**Employment**				
Full-time	480 (36%)	415 (37%)	65 (31%)	0.21
Part-time	121 (09%)	96 (9%)	25 (12%)	
Retired	566 (43%)	477 (43%)	89 (43%)	
Unemployed	162 (12%)	133 (12%)	29 (14%)	
**Medical comorbidities**				
**History of heart failure**				
Yes	99 (7%)	75 (7%)	24 (12%)	0.014
No	1,230 (93%)	1,046 (93%)	184 (88%)	
**History of chronic renal insufficiency**				
Yes	99 (7%)	66 (6%)	33 (16%)	<0.001
No	1,230 (93%)	1,055 (94%)	175 (84%)	
**History of previous stroke or TIA**				
Yes	273 (21%)	209 (19%)	64 (31%)	<0.001
No	1,056 (79%)	912 (81%)	144 (69%)	
**History atrial flutter or fibrillation**				
Yes	179 (13%)	147 (13%)	32 (15%)	0.38
No	1,150 (87%)	974 (87%)	176 (85%)	
**Smoker**				
Yes	290 (22%)	256 (23%)	34 (16%)	0.037
No	1,039 (78%)	865 (77%)	174 (84%)	
**Hypertension**				
Yes	1,004 (76%)	839 (75%)	165 (79%)	0.17
No	325 (24%)	282 (25%)	43 (21%)	
**Diabetes**				
Yes	417 (31%)	338 (30%)	79 (38%)	0.025
No	912 (69%)	783 (70%)	129 (62%)	
**Dyslipidemia**				
Yes	598 (45%)	504 (45%)	94 (45%)	0.95
No	731 (55%)	617 (55%)	114 (55%)	
**Stroke severity**				
NIHSS, Median (Interquartile Range)	3 (5)	2 (4)	4 (7)	<0.0001
NIHSS ≤ 3	799 (60%)	698 (62%)	101 (49%)	0.0002
NIHSS >3	530 (40%)	423 (38%)	107 (51%)	
**Ischemic stroke etiology (TOAST)**				
Large-artery atherosclerosis	190 (14%)	163 (14%)	27 (13%)	0.27
Cardio-embolism	238 (18%)	205 (18%)	33 (16%)	
Small vessel disease	206 (16%)	182 (16%)	24 (12%)	
Stroke of other undetermined etiology	43 (3%)	34 (3%)	9 (4%)	
Cryptogenic stroke	393 (30%)	322 (29%)	71 (34%)	
Missing or non-ischemic stroke	259 (19%)	215 (19%)	44 (21%)	
**Stroke type**				
Ischemic	1,210 (91%)	1,025 (92%)	185 (89%)	0.23
Hemorrhagic	118 (9%)	95 (8%)	23 (11%)	
**Neighborhood characteristics**				
**Region of FL**				
North	233 (18%)	204 (18%)	29 (14%)	0.45
Panhandle	32 (2%)	26 (2%)	6 (3%)	
South	490 (37%)	407 (36%)	83 (40%)	
West Central	574 (43%)	484 (43%)	90 (43%)	
**Neighborhood has at least 1 rehab facility**				
No	947 (71%)	793 (71%)	154 (74%)	0.33
Yes	382 (29%)	328 (29%)	54 (26%)	
**Neighborhood has** ≥**1 hospital**				
No	0856 (64%)	0723 (64.50%)	0133 (64%)	0.88
Yes	0473 (36%)	0398 (35.50%)	0075 (36%)	
**Median clinic density**				
Below median	0522 (39%)	0448 (40%)	0074 (36%)	0.23
Above median	0807 (61%)	0673 (60%)	0134 (64%)	
**RUCA score 1 vs. the rest**				
Other scores	184 (14%)	163 (15%)	21 (10%)	0.09
RUCA = 1	1,145 (86%)	958 (85%)	187 (90%)	

Red color used for statistically significant *p*-values.

Among the 1,329 stroke patients (56% male; median age: 64 years; 51% White, 23% Black, 22% Hispanic, and 4% other races), 208 experienced readmission or death within 90 days post-discharge. This included 189 patients who were readmitted (3 of whom died during the 90-day period) and 19 who died without readmission.

Premorbid conditions including heart failure, chronic renal insufficiency, diabetes, previous stroke or TIA, stroke severity, and smoking history were significantly associated with 90-day readmission or death (Table 2).

In multivariable logistic regression, only neighborhood **Factor 1**, characterized by high population density, lower neighborhood socioeconomic status, and greater urbanization, with higher proportions of Hispanic residents among correlated demographic features, was associated with higher odds of the 90-day composite of readmission or death: after adjustment for demographics and individual socioeconomic characteristics (Model 1), Factor 1 had an OR = 1.18 (*p* = 0.04). This association persisted and modestly strengthened with additional adjustment for cardiovascular comorbidities, prior stroke, smoking history, and NIHSS (Model 2: OR = 1.20; *p* = 0.03). Factors 2 through 4 showed no association with the outcome in either model: Factor 2 (high-SES White-dominant); Factor 3 (high-SES, lower business density, and Hispanic-dominant); and Factor 4 (areas with high accessibility to rehabilitation and clinical or hospital services; Table 3).

**Table 3:** Logistic regression analysis of each factor with death or readmission within 90-days post-discharge in stroke survivors.

**Factor**	**Model 1: demographics (age, sex, race), socioeconomic status (person they live with, social support size, health insurance type, education)**			**Model 2: Model 1** + **cardiovascular comorbidities and previous stroke, smoking history, stroke severity (NIHSS), discharge destination**		
	**Odds ratio**	**95% Wald CI**	***p*** **-value**	**Odds ratio**	**95% Wald CI**	***p*** **-value**
Factor 1	1.18	1–1.39	0.04	1.2	1.02–1.41	0.03
Factor 2	0.98	0.82–1.15	0.77	1.01	0.84–1.20	0.92
Factor 3	1.06	0.88–1.28	0.53	1.11	0.91–1.35	0.30
Factor 4	0.94	0.80–1.1	0.44	1.08	0.92–1.28	0.38

Factor 1: crowded, Hispanic dominant, low SES and high business densities; factor 2: low population, white dominant, high-SES and low business densities; factor 3: crowded, Hispanic dominant, high-SES but low business densities; factor 4: high rehab and clinical/hospital services accessibility. NIHSS has 4 categories: (1) 0–4= minor stroke (*n* = 902, 67.9%); (2) 5–15 = moderate stroke (*n* = 350, 26.3%); (3) 16–20 = moderate to severe stroke (*n* = 46, 3.5%); and (4) 21 and above = severe stroke (*n* = 31, 2.3%). Red color used for statistically significant *p*-values.

In sensitivity analyses, associations with neighborhood Factor 1 were attenuated and did not reach statistical significance when outcomes were examined separately (readmission alone and death alone; Supplementary Tables 1, 2).

## Discussion

In this analysis we found that neighborhood characteristics are significantly associated with 90-day mortality and readmission among stroke survivors. We found that living in low-socioeconomic-status (SES), and highly urbanized neighborhoods was significantly associated with an increased likelihood of adverse outcomes within 90 days post-discharge. These findings highlight the need to consider broader social and environmental factors, particularly neighborhood socioeconomic status (NSES), when assessing stroke outcomes- regardless of individual SES or stroke severity. The significant association of factor 1 characterized by low neighborhood socioeconomic status, high population density, and intense urbanization (including higher business densities and RUCA score) with a 20% increased risk of adverse outcomes post-stroke, underscores the importance of structural neighborhood disadvantage, as well as the impact that higher urbanization, population and business densities can have on exacerbating patients' challenges and vulnerability after hospital discharge to these areas. These challenges may be related to lower access to healthier lifestyle choices (e.g., limited access to supermarkets, reduced availability of fresh products, lower neighborhood density of parks, gyms, community health clubs) as well as higher density of fast-food establishments, alcohol and tobacco outlets captured within factor 1 neighborhoods. Prior studies have demonstrated links between the density of harmful business outlets and poor health outcomes, including higher substance use rates (Wheeler et al., 2022; Shortt et al., 2015), and difficulty quitting these substances with misuse potential (e.g., alcohol, tobacco, and other drugs) (Finan et al., 2019; Marsh et al., 2021). Moreover, there is a direct association between higher fast food densities in neighborhood and risk of stroke (Morgenstern et al., 2009), stroke mortality (Mazidi and Speakman, 2018), and increased cardiovascular risk (Liang et al., 2022). Furthermore, the higher level of urbanization, population and business densities reflected in factor 1 can increase exposure to air pollutants due to elevated traffic and commuting activity. While a significant association between air pollution and risk of stroke (Swieczkowski et al., 2024; Liu et al., 2023; Avellaneda-Gomez et al., 2022; Shin et al., 2019), stroke severity (Wing et al., 2017), and mortality rates (Liao et al., 2022) have been reported, their impact on short-term 90-day outcomes post-stroke is less established.

Lower NSES is a well-documented predictor of higher risk of stroke and cardiovascular diseases (Malla et al., 2024; Ortiz-Whittingham et al., 2023; Jensen et al., 2023; Xiao et al., 2022; Uddin et al., 2022; Kim et al., 2021), mortality (Zhang et al., 2022), and readmission (Lusk et al., 2024), as well as poorer functional recovery post-stroke (Cote et al., 2024). Low NSES is additionally associated directly with epigenetic biomarkers of aging as predictors of cardiovascular diseases and mortality (IFN gamma, PCSK9, HDL subspecies) (Ortiz-Whittingham et al., 2023).

Although some studies have identified racial and ethnic composition as predictors of stroke prevalence (Hu et al., 2021; Ji et al., 2020), caution is warranted when interpreting these findings in the context of this study. The significance of Hispanic dominant areas in Factor 1, given Florida's large Hispanic population, may reflect a geographic proxy for southern neighborhoods rather than a direct association with race or ethnicity. Notably, Hispanic-dominant neighborhoods with higher SES (Factor 3) were not associated with worse outcomes, further suggesting that socioeconomic and environmental factors, rather than race or ethnicity, drive these disparities. Thus, these findings should not be interpreted as definitive evidence of the impact of neighborhood racial and ethnic composition on stroke prognosis.

### Implications for interventions

The significant association between Factor 1 and poor outcomes underscores the need for targeted interventions in neighborhoods with low SES and high population and business densities.

- Promoting healthy behaviors: initiatives could be aimed at improving access to healthcare, reducing the impact of harmful businesses, increasing availability of affordable, nutritious food, and providing educational resources about tobacco and alcohol cessation. For example, programs that focus on promoting healthy behaviors and secondary stroke prevention, using focused educational interventions (Towfighi et al., 2021, 2017; Boden-Albala et al., 2019) could play a critical role in improving long-term stroke outcomes.
- Enhancing care and service access: providing culturally and linguistically appropriate post-discharge care, particularly for populations with limited English proficiency, may improve adherence to rehabilitation and follow-up care. Additionally, social support services in lower SES areas could mitigate the risk of death and readmission post-stroke.
- Improving neighborhood environments: policies to reduce air pollution, expand access to green spaces, improve housing, expand employment opportunities, improve educational access, and provide affordable housing—all may have a direct positive impact on stroke recovery outcomes.

### Limitations and future research

While our findings suggest that lower access to healthier lifestyle resources and higher density of fast-food establishments, alcohol, and tobacco outlets are associated with adverse 90-day outcomes, it is important to acknowledge that inferring causality in this context may be premature. These environmental exposures likely reflect longstanding neighborhood conditions that predate the stroke event and may have contributed to individuals' premorbid health profiles and comorbidities, which in turn increase their vulnerability to readmission or death after discharge. Therefore, the observed associations may not reflect the immediate impact of post-discharge neighborhood exposures, but rather the cumulative effects of chronic environmental and socioeconomic disadvantage over time. This highlights the potential for residual confounding and underscores the need for longitudinal studies with more granular, time-sensitive exposure data to disentangle these effects and clarify temporal relationships.

When outcomes were examined separately, either as readmission alone or death alone, associations with neighborhood disadvantage were attenuated and did not reach statistical significance. This attenuation likely reflects reduced statistical power and outcome heterogeneity, and suggests that neighborhood-level disadvantage may be more strongly related to severe post-stroke outcomes captured by the composite endpoint than to either component alone.

This study used ZIP+4 codes as proxies for neighborhoods, which may not fully capture the social and physical dynamics influencing health outcomes. Future research should explore alternative geographic units of analysis (e.g., census block groups) and consider additional neighborhood factors which have been shown to be linked to stroke risk profile, such as environmental hazards, particulate matter, crime rate, walkability, and access to green spaces (Liao et al., 2022; Lang et al., 2022; Yitshak-Sade et al., 2019; Yang et al., 2022; Ruiz et al., 2021), that may also influence stroke recovery process post discharge. Additionally, reliance on self-reported data for individual factors like social support and living arrangement may introduce bias to study results due to misclassification.

This analysis was restricted to stroke survivors discharged to home or inpatient rehabilitation with complete 30- and 90-day follow-up, reflecting the study's focus on post-discharge behavioral modifications and transitional care processes that require sufficient functional capacity for independent participation. Patients discharged to skilled nursing facilities or long-term acute care hospitals are typically more medically complex and receive structured, provider-directed care, limiting the interpretability of individual-level behavioral measures in this group. Consequently, the observed associations may underestimate the true magnitude of neighborhood-level disadvantage on post-discharge outcomes and limit generalizability to the most medically complex stroke survivors.

Finally, while we adjusted for key individual and clinical factors, there may be other unmeasured variables, such as behavioral modifications post discharge (Johnson et al., 2024), that deserve attention in future studies. Longitudinal studies with more detailed information on post-discharge care and community support services could provide further clarity on the mechanisms linking neighborhood environments to stroke outcomes.

## Conclusion

Our findings highlight the significant role of neighborhood socioeconomic factors in shaping stroke recovery. Structural neighborhood disadvantage, characterized by low socioeconomic resources, high population and business density, and intense urbanization, was independently associated with increased risk of 90-day readmission or death following stroke. Public health policies and community interventions that address social determinants of health are essential to improving outcomes for stroke survivors, particularly those in vulnerable neighborhoods.

## Data Availability

The data analyzed in this study is subject to the following licenses/restrictions: the Florida Stroke Registry (FSR) utilizes data from Get With The Guidelines-Stroke^®^ (GWTG-S), collected primarily for quality improvement. Researchers seeking access to this data must submit a research proposal through http://www.heart.org/qualityresearch. Proposals are reviewed by the GWTG-S and FSR advisory and publication committees upon reasonable request. Requests to access these datasets should be directed to http://www.heart.org/qualityresearc.
