# Supplementary figures and images for: Neighborhood economic and demographic landscape as predictors of 90-day outcomes post-stroke hospitalization
Source: Front Stroke. 2026 Mar 12;5:1738822. doi: 10.3389/fstro.2026.1738822 (PMC13017339; doi:10.3389/fstro.2026.1738822)

Supplemental figure 1: Scree Plot of Eigenvalues:


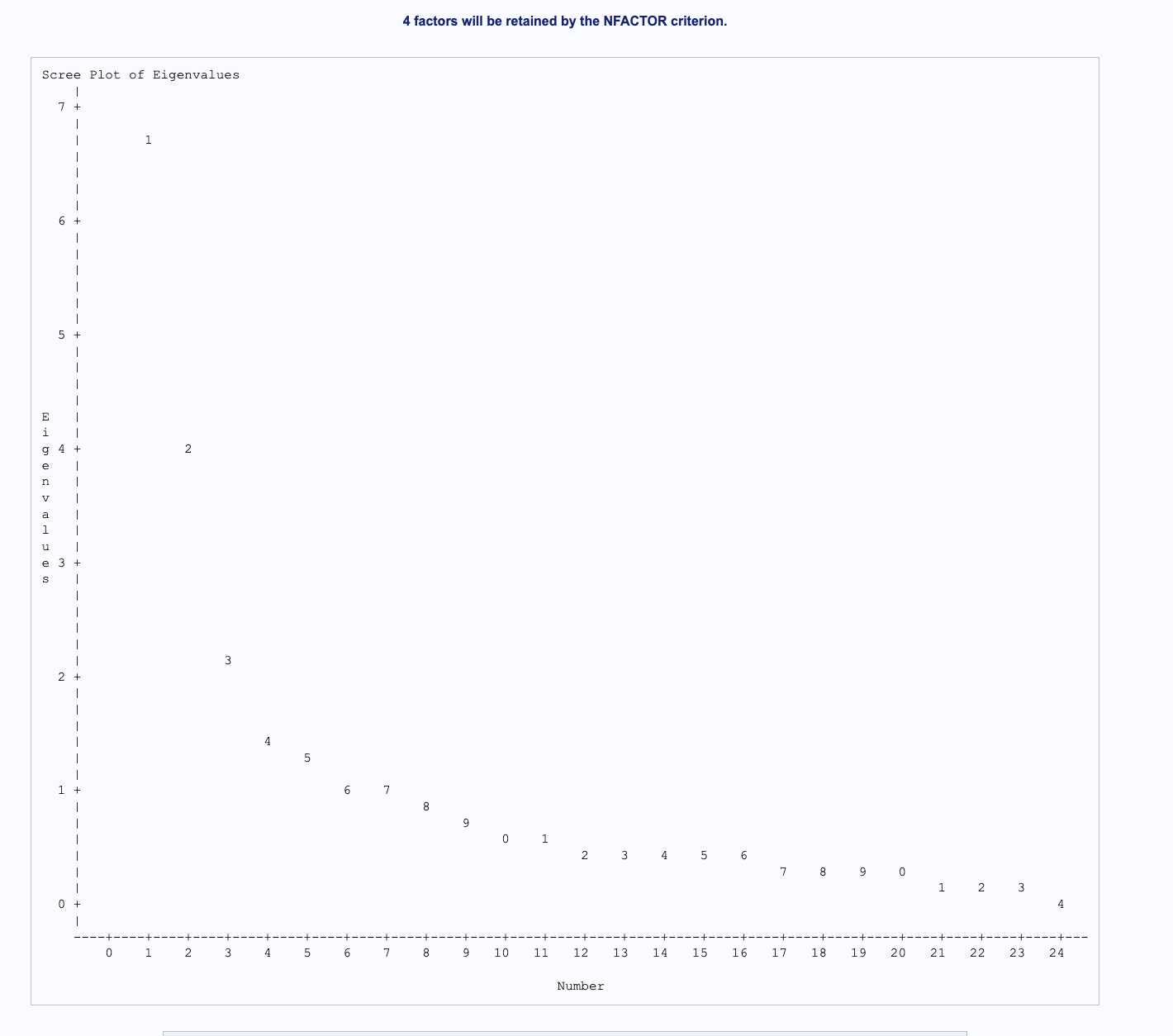

Supplement: Supplementary file 1 [file Supplementary_file_1.docx]
