# Supplementary material for: Neighborhood economic and demographic landscape as predictors of 90-day outcomes post-stroke hospitalization
Source: Front Stroke. 2026 Mar 12;5:1738822. doi: 10.3389/fstro.2026.1738822 (PMC13017339; doi:10.3389/fstro.2026.1738822)
Supplement: Supplementary file 3 [file Table_1.docx]

Supplemental Tables

**Readmission Only**

| Supplemental Table 1: logistic regression analysis of each factor with readmission only within 90-days post-discharge in stroke survivors | | | | | | |
| --- | --- | --- | --- | --- | --- | --- |
|  | **Model 1: demographics (age, sex, race), socioeconomic status (person they live with, social support size, health insurance type, education)** | | | **Model 2: Model 1 + cardiovascular comorbidities and previous stroke, smoking history, stroke severity (NIHSS)** | | |
|  | **Odds Ratio** | **95% Wald CI** | **p-value** | **Odds Ratio** | **95% Wald CI** | **p-value** |
| Factor 1 | 1.12 | 0.98-1.32 | 0.15 | 1.15 | 0.97-1.35 | 0.10 |
| Factor 2 | 1.03 | 0.86-1.23 | 0.73 | 1.04 | 0.87-1.25 | 0.67 |
| Factor 3 | 1.13 | 0.93-1.37 | 0.21 | 1.15 | 0.94-1.40 | 0.17 |
| Factor 4 | 1.08 | 0.92-1.28 | 0.34 | 1.05 | 0.89-1.25 | 0.55 |
| Factor 1: crowded, Hispanic dominant, low SES and high business densities  Factor 2: low population, white dominant, high-SES and low business densities  Factor 3: crowded, Hispanic dominant, high-SES but low business densities  Factor 4: high rehab and clinical/ hospital services accessibility | | | | | | |

**Death Only**

| Supplemental Table 2: logistic regression analysis of each factor with death or readmission within 90-days post-discharge in stroke survivors | | | | | | |
| --- | --- | --- | --- | --- | --- | --- |
|  | **Model 1: demographics (age, sex, race), socioeconomic status (person they live with, social support size, health insurance type, education)** | | | **Model 2: Model 1 + cardiovascular comorbidities and previous stroke, smoking history, stroke severity (NIHSS)** | | |
|  | **Odds Ratio** | **95% Wald CI** | **p-value** | **Odds Ratio** | **95% Wald CI** | **p-value** |
| Factor 1 | 1.58 | 0.95-2.64 | 0.08 | 1.54 | 0.91-2.63 | 0.11 |
| Factor 2 | 0.86 | 0.53-1.39 | 0.53 | 0.84 | 0.51-1.39 | 0.50 |
| Factor 3 | 0.83 | 0.45-1.53 | 0.55 | 0.87 | 0.45-1.69 | 0.69 |
| Factor 4 | 1.55 | 0.82-2.92 | 0.18 | 1.47 | 0.76-2.84 | 0.26 |
| Factor 1: crowded, Hispanic dominant, low SES and high business densities  Factor 2: low population, white dominant, high-SES and low business densities  Factor 3: crowded, Hispanic dominant, high-SES but low business densities  Factor 4: high rehab and clinical/ hospital services accessibility | | | | | | |
